# Alternating Binary Multilayers of Alkanethiol-Modified Gold Nanoparticles and Quantum Dots with Artificial Three-Dimensional Structures and Rational Photoluminescence

**DOI:** 10.1021/acsami.5c02956

**Published:** 2025-05-02

**Authors:** Rina Sato, Hideyuki Mitomo, Yuto Kajino, Masaki Matsubara, Takehiro Yachi, Megumi Suyama, Kaoru Tamada, Kiyoshi Kanie

**Affiliations:** † Institute of Multidisciplinary Research for Advanced Materials, 13101 Tohoku University, 2-1-1 Katahira, Aoba-ku, Sendai, Miyagi 980-8577, Japan; ‡ Research Institute for Electronic Science, 12810 Hokkaido University, Kita 21, Nishi 10, Kita-Ku, Sapporo, Hokkaido 001-0021, Japan; § Institute for Materials Chemistry and Engineering, 12923 Kyushu University, 744 Motooka, Nishi-Ku, Fukuoka 819-0935, Japan; ∥ International Center for Synchrotron Radiation Innovation Smart, 13101 Tohoku University, 2-1-1 Katahira, Aoba-ku, Sendai, Miyagi 980-8577, Japan; ⊥ National Institute of Technology, Sendai College, Nodayama-Shiote 48, Medeshima, Natori, Sendai, Miyagi 981-1239, Japan

**Keywords:** gold nanoparticles, quantum dots, multilayer, binary nanoparticle superlattice, Langmuir−Schaefer method

## Abstract

The formation of periodic arrangements of two types of nanoparticles (NPs), i.e., binary NP superlattices (BNSLs), provides a versatile approach to control their physical properties through interparticle interactions. However, achieving highly ordered BNSLs is still challenging because of the difficulty of combining two distinct NPs without phase segregation. In this work, plasmonic Au NPs and CdS quantum dots (QDs) modified with dodecanethiol ligands were assembled into binary multilayered structures by alternately laminating their monolayers onto a single substrate. Grazing-incidence small-angle X-ray scattering analysis confirmed the long-range ordered arrangement of the NPs, demonstrating the successful fabrication of 3D structures with Au NPs and CdS QDs. The formation of close-packed monolayers and entropic stabilization by surface ligands were identified as key factors in artificially constructing such 3D arrays via multilayering. The formation of defect-free BNSLs was also optically identified by a systematic increase in the intensities of the photoluminescence (PL) and plasmon extinction intensities, as well as perfect control of respective band positions. Based on finite-difference time-domain simulations, this PL behavior was attributed to the formation of a layered superstructure with a homogeneous dielectric function in the repeat unit of a Au NP-CdS QD-Au NP layer. This work demonstrates that simple alternative lamination of NP monolayers offers a facile yet effective strategy to obtain BNSLs with well-defined physical properties for broader application of NP array-based materials.

## Graphical abstract

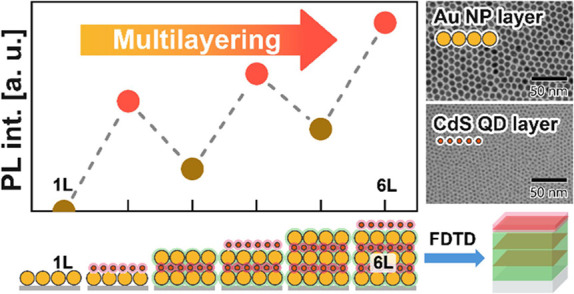

## Introduction

Gold nanoparticles (Au NPs) and quantum dots (QDs) have been extensively studied in recent years for their characteristic optical properties.^1–5^ Their physical properties, such as localized surface plasmon resonance (LSPR) and photoluminescence (PL), can be tuned by controlling not only their size, shape, or composition but also the interparticle distance since inorganic NPs readily interact with neighbors. For example, for Au NPs, near-field plasmon coupling is induced between adjacent NPs, which causes a significant shift in their LSPR extinction wavelength toward the longer side compared with the isolated state.^6,7^ The hotspots generated between proximal Au NPs remarkably increase the Raman scattering intensity, making these NPs useful for molecular detection. Additionally, the rates of energy and electron transfer are strongly affected by the interparticle distance of QDs.^8,9^

The formation of 1D, 2D, or 3D arrays, in which NPs are building blocks, enables control over the physical properties of NPs derived from the interparticle interactions.^10–14^ Beyond spherical NPs, arrangement of NPs with anisotropic shapes, such as rods or plates, can provide unusual properties by utilizing “faces” or “vertices” not present in spherical particles.^15–17^ The self-assembly of inorganic NPs modified with organic ligands on a water surface represents a groundbreaking method for obtaining 2D superlattices with controlled interparticle distance in a quick and suitable manner.^18–20^ The close-packed array of NPs formed on the water surface can be directly transferred onto a hydrophobic substrate, and this process can be repeated to achieve lamination on the same substrate.^21^ This multilayering technique enables facile formation of 3D structures with strictly defined in-plane and out-of-plane distances using NPs, as well as expected novel functionalities and optical interactions between the NPs and various substrates.^22^

When such NP assemblies are extended from unary to binary or ternary systems,^23–32^ they have greater potential because of their designable functions, as the optical, magnetic, or electrochemical properties of NPs can be synergistically combined depending on their regular assembly structure.^26,27^ For example, Murray et al. demonstrated that the magnetic resistance properties of a binary NP superlattice (BNSL) composed of Fe_3_O_4_ and FePt NPs could be drastically tuned via the NP arrangement.^28^ Therefore, the formation of BNSLs leads to control over interparticle interactions and provides innovative advanced nanomaterials. In numerous studies, self-organization of organic ligand-coated NPs, facilitated by capillary effects during solvent evaporation, has been a popular method for forming NP superlattices.^12,29–33^ In general, the dominant driving force for BNSL formation is still unclear even though various interactions, such as the Coulomb force, van der Waals attraction between metal cores and steric repulsion between organic ligands, have been discussed. Spontaneous formation of 3D structures at the micron scale via these techniques requires slow evaporation over several hours, sometimes more than 12 h, under reduced pressure and moderately high-temperature conditions. Additionally, precisely defined mixing ratio and size ratio of NPs are essential factors not for forming a phase-segregated structure but for creating long-range ordered binary systems.

In this work, we tethered a binary multilayer by alternately laminating monolayers of Au NPs and CdS QDs modified with dodecanethiol (DT) ligands (Figure 1a) via the Langmuir–Schaefer method.^34^ We first prepared the NP monolayer through self-assembly on a water surface, with rapid evaporation of the organic solvent under ambient temperature and pressure. The concentration and amount of the droplet were determined to obtain a close-packed 2D arrangement without continuous compression. Inorganic NPs were coated with hydrophobic organic ligands so that we could laminate the NP monolayers several times through the hydrophobic interactions at the interfaces of the NP layers (Figure 1b). This multilayering method allows effortless and orderly combination of two types of NPs, which endures, thus avoiding phase segregation. Additionally, the multilayered superlattice, whose structure is defined by the thickness of the NP monolayer, provides a model for evaluating the array structure and synergistic interparticle interactions. We clarified the spatial arrangement of neighboring NPs in BNSLs constructed from spherical Au NPs and CdS QDs and investigated the correlation between the NP superstructure and the PL properties of QDs via experimental and numerical simulations using the finite-difference time-domain (FDTD) method.

**Figure 1:**
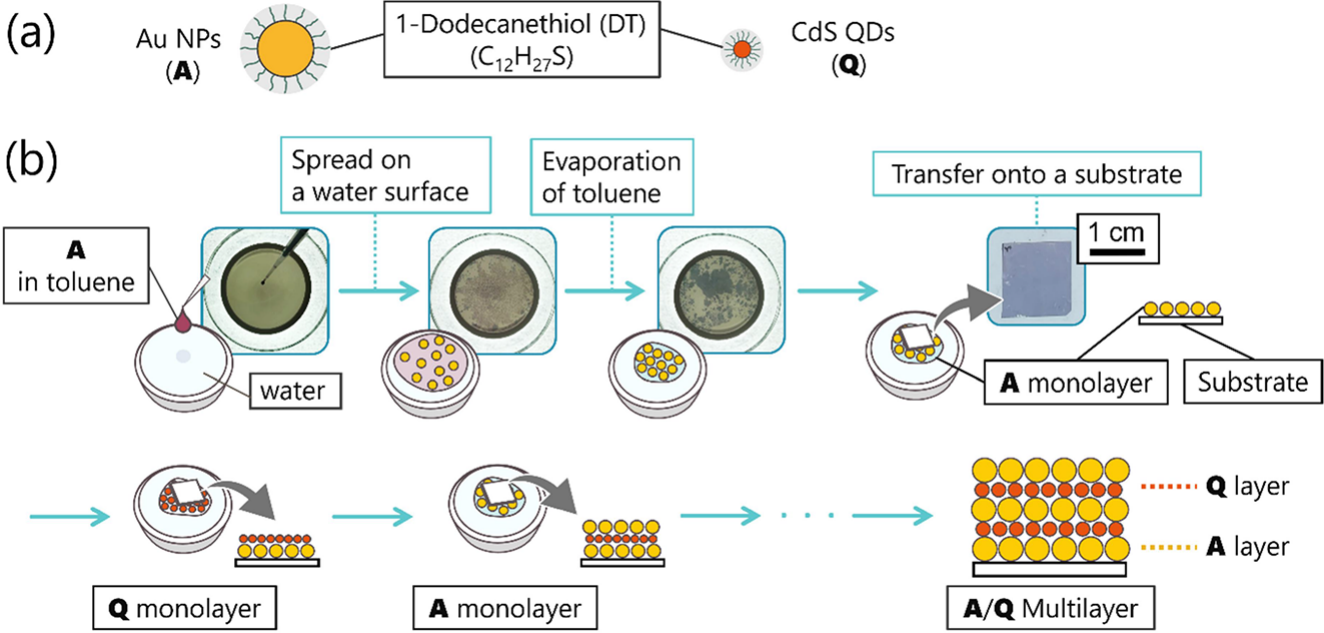
(a) Schematic illustrations of DT-modified NPs. (b) Illustrations and snapshots during the process of multilayer formation by laminating NP monolayers spread on a water surface. **A** and **Q** are represented as yellow and red spheres, respectively.

## Experiment

### Chemicals

Hydrogen tetrachloroaurate­(III) tetrahydrate was purchased from Wako Pure Chemical Industries. 1-DT, borane-*tert*-butylamine complex, and 1,1,3,3-tetramethylthiourea were supplied by Sigma–Aldrich. Octyl ether was purchased from Tokyo Chemical Industry. Cadmium acetate dihydrate and tetramethylammonium hydroxide for the synthesis of cadmium stearate were also purchased from Wako Pure Chemical Industries. All the chemicals were used without further purification.

### Preparation of Nanoparticle Monolayers and Multilayers

Initially, DT-modified Au NPs (**A**) and CdS QDs (**Q**) were synthesized following previous methods^35,36^ with some modifications. In a typical procedure, hydrogen tetrachloroaurate­(III) tetrahydrate (0.125 mmol) was initially dissolved in 8.0 mL of tetrahydrofuran (THF), to which DT (0.085 mmol) was added, followed by stirring for 3 h at room temperature. Borane-*tert*-butylamine complex (0.250 mmol) dissolved in 2.0 mL of THF was subsequently added to the mixture in one portion to reduce Au­(III) at 8 °C. The solution was centrifuged and washed with hexane/ethanol to remove excess DT, and DT-modified Au NPs (**A**) were finally obtained. The coating of the NPs with DT allows them to be dispersed in various nonpolarized organic solvents, such as chloroform, toluene, and hexane. **A** and **Q** were dispersed in these solvents and then dropped onto the convex surface of water within a Teflon container (6 cm in diameter). As a result, the dispersion spontaneously spread across the surface of the water and formed a monolayer as the organic solvent evaporated. Note that the concentration of the NP dispersion and the droplet amount were determined by assuming that the spherical NPs spread to the same area on the water surface. The concentration of the NP dispersion plays a key role in spontaneous spreading on the water surface. The surface tension between a nonpolar organic solvent and water can be decreased by including surfactant-modified NPs in an organic solvent, leading to the formation of a monolayer structure. This means that NP dispersions at low concentrations cannot spontaneously spread; at the same time, too high a concentration can suppress the movement of NPs owing to the relatively high viscosity.^20^ The NP monolayer could be transferred onto hydrophobic substrates (e.g., quartz glass, silicon wafers, or TEM grids) several times, which allowed the monolayers to be laminated and a multilayered structure to be prepared on the substrate. **A**/**Q** was finally obtained by alternately laminating monolayers of **A** and **Q** on a glass slide substrate.

### Characterizations

The morphologies of the obtained NPs and mono/multilayers were characterized by transmission electron microscopy (TEM) (HITACHI H-7650, accelerating voltage of 100 kV, emission current of 20 μA) and scanning transmission electron microscopy (STEM) (FEI-Company, Titan3^TM^ 60-300). Information on the NP arrangement structure was obtained via grazing-incidence small-angle X-ray scattering (GI-SAXS) (SPring-8 BL03XU) measurements. The extinction spectra of the NPs and their mono/multilayers were evaluated via ultraviolet/visible (UV/vis) spectroscopy (HITACHI, U-3900). In addition, PL spectra were measured via fluorescence microscopy (HITACHI, F-2700, excitation wavelength of 375 nm) for the NP dispersion and via an epifluorescence microscope system with a CCD camera and a spectrograph (Olympus, DP72, excitation wavelength of 400 nm) for the mono/multilayers. Note that emission wavelengths shorter than 450 nm were cut off by a filter. All the measurements for characterization of optical properties were performed every laminating and not repeated (*n* = 1).

### FDTD Simulations

FDTD simulations were conducted with Poynting for Optics software (Fujitsu, Japan) to discuss the emission system based on alternating multilayer formation.^37,38^ In the FDTD simulation model, a monolayer of an **A** or **Q** layer was treated as a homogeneous dielectric medium (effective medium approximation) instead of an NP assembly.^38^ The superlattice structure with a **Q** layer sandwiched between two **A** layers, named the **AQA** layer, was also treated in the same way, and further details are presented later. For the optical constants of the **Q** layer, those of bulk CdS in the region without interband absorption were used, giving a refractive index of 2.25 and no absorption.^39^ The optical parameters for the **A** layer were determined by fitting the absorption spectrum with two Lorentz functions, which were attributed to the interband absorption of gold (resonance energy *ℏ*ω_0_ = 2.06 eV, damping constant *ℏ*γ = 0.05 eV, plasma frequency *ℏ*ω_p_ = 2.47 eV) and LSPR absorption (*ℏ*ω_0_ = 5.72 eV, *ℏ*γ = 13.2 eV, *ℏ*ω_p_ = 11.4 eV), respectively.^38^ The emission from the **Q** layer was modeled by a plane light source composed of a modulated Gaussian function with a width of 5 fs and a period of 1.5 fs, corresponding to a center wavelength of 454 nm, placed in the center of the **Q** layer. The **A** and **Q** layers on a glass substrate (refractive index of 1.5) were placed on the *X*–*Y* plane under periodic boundary conditions in both the *X* and *Y* directions. A nonuniform mesh with a grid size of 1–2 nm was employed. The PL intensity of the model was determined using a point light detector positioned 350 nm above the glass substrate in the *Z* direction.

## Results and Discussion

### Formation of NP Monolayers

Figure 2a shows a photograph, a TEM image, and a fast Fourier transform (FFT) image of the monolayer obtained from **A** using toluene as the solvent. As shown in Figure 2a,b­(i), homogeneous films spread across the water surface at the centimeter scale. According to the TEM image in Figure 2a­(ii), **A** was monodispersed and spherical in shape, and the monolayer consisted of a highly uniform hexagonal array with a few defects. The size and intersurface distance of **A** calculated from the TEM image were 9.4 ± 0.2 and 1.5 ± 0.2 nm, respectively. Additionally, the FFT image of the **A** monolayer shown in Figure 2a­(iii) exhibited almost a spot-like pattern, indicating a highly periodic structure of **A** with a specific orientation.

**Figure 2:**
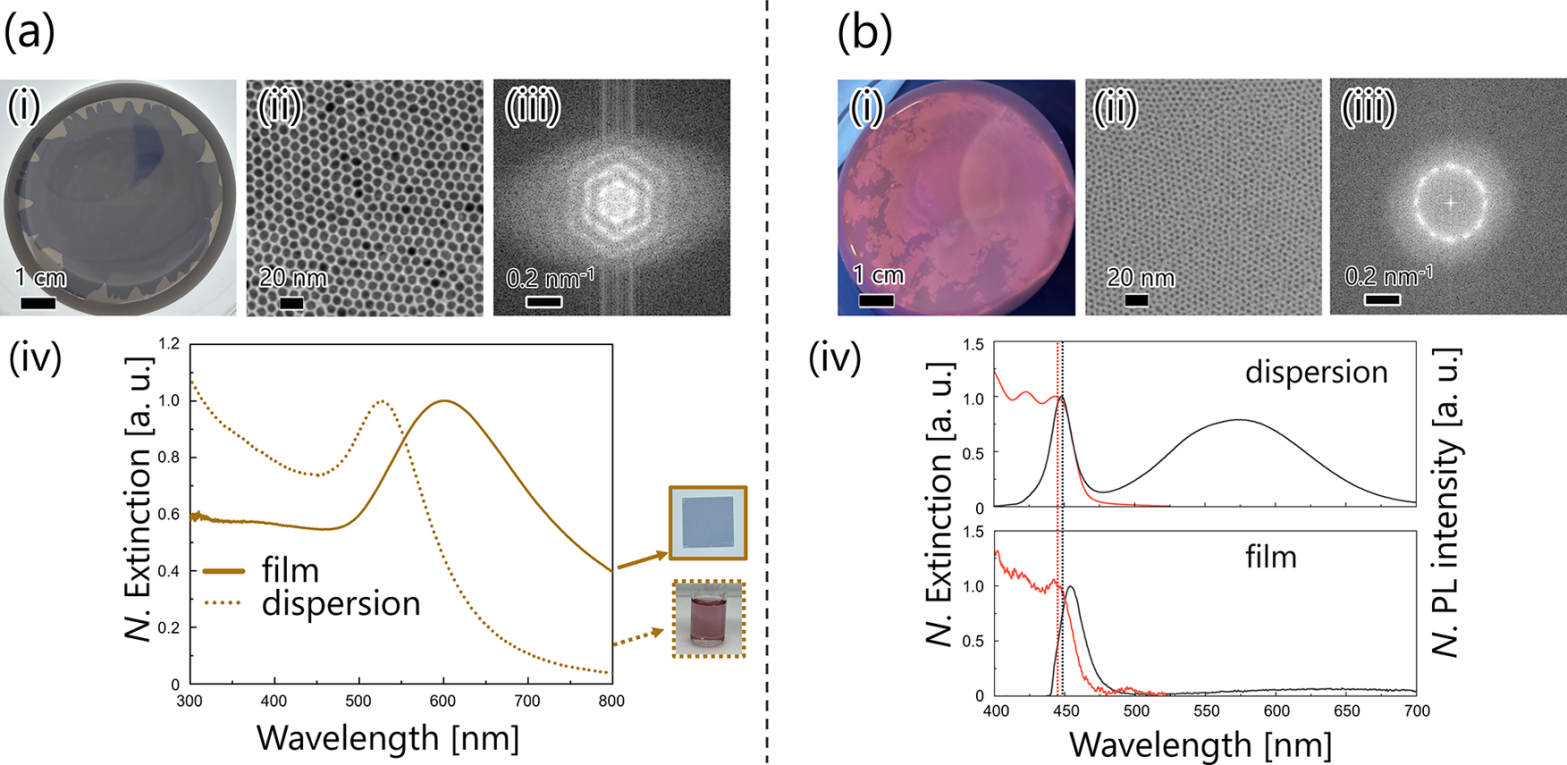
Characterization of (a) **A** or (b) **Q** monolayer. (i) Photograph of the monolayer spread on a water surface. Photographs of each monolayer taken under (a)­(i) transmission light from the bottom of the container or (b)­(i) irradiation by UV light at a wavelength of 365 nm. (ii) TEM image and (iii) FFT image acquired from (ii). The TEM observation was carried out on the monolayer vertically transferred from the water surface onto a TEM grid. (iv) UV/vis extinction spectra and PL spectra (only the black line in (b)­(iv)) of the dispersion and film states. (a)­(iv) Photographs of a film on a glass slide (1.3 cm square) or a dispersion of **A** in toluene, presented in the solid or dotted frame, respectively. The extinction and PL intensities were normalized to the values at the LSPR, band-edge absorption, and band-edge emission wavelengths. In addition, the baseline was subtracted from the extinction spectra of both the film and dispersion of **Q**.

The monolayer of **A** showed a vibrant blue color, which was distinct from the well-known reddish color of the dispersed state. As shown in Figure 2a­(iv), in the monolayer state of **A**, the extinction wavelength derived from LSPR shifted toward a longer wavelength by 65 nm compared to that in the dispersed state. In the homogeneous dispersion, the NPs were separated enough to absorb light at the specific wavelengths derived from an individual NP. In the monolayer state, the distance between neighboring NPs reached 1.5 nm, generating a strongly enhanced electric field around the Au NPs and shifting the extinction wavelength toward the lower energy side, i.e., the longer wavelength side.

The appearance of such monolayers is thus heavily affected by the overall arrangement of the Au NPs. We observed a monolayer with numerous grain boundaries formed by Au NPs with a diameter of 5.5 nm when chloroform was used as the solvent. The resulting monolayer was purple in color (Figure S1). Thus, both less plasmon coupling between domains and strong plasmon coupling inside each domain occurred, and broad absorption ultimately appeared at 500–650 nm (Figure S2).

After the NPs were deposited onto the water surface, the NPs in the organic solvent were initially randomly distributed across the surface. As the solvent evaporated, these NPs aggregated into discrete islands with a hexagonal arrangement, and these islands eventually gathered via interactions among DT ligands at island interfaces, forming a long-range NP-monolayer.^40–42^ Basically, ligands can extend around an NP core through solvent molecules entering between ligand chains if the Hansen solubility parameters (HSPs) of the ligand and solvent are close.^40,43^ The HSPs of chloroform and toluene are 19.0 and 18.7, respectively, which are both close to that of DT (17.6). Chloroform has a higher water solubility and a lower boiling point than toluene, which results in a faster evaporation rate after it is dropped on the water surface. Therefore, a polydomain structure with numerous grain boundaries was observed in the monolayer when using chloroform as the solvent, which maintained the flexibility of the ligands and evaporated too quickly to ensure sufficient time for reduction of the boundaries between domains through ligand interactions. Toluene evaporated so slowly on the water surface that a regular NP arrangement with fewer domains could be formed. In particular, the solvation repulsive interaction has been reported to facilitate the formation of island-like aggregates and affect the evaporation rate of organic solvents around NPs.^44^ Indeed, confirmation of the formation of a solid film with the naked eye took approximately several seconds when using toluene, despite this formation only taking moments with chloroform.

Accordingly, toluene was also employed for the fabrication of **Q** monolayers. The size and intersurface distance of **Q** were 3.1 ± 0.5 and 2.8 ± 0.1 nm, respectively (Figure 2b­(ii)). A long-range ordered hexagonal array, supported by the FFT pattern in Figure 2b­(iii), was observed, similar to the **A** monolayer. In terms of the optical properties, the **Q** monolayer exhibited a fluorescent yellow color under UV light at 365 nm when observed with the naked eye (Figure 2b­(i)) and was transparent under ambient light. Notably, the UV light at 365 nm made the **Q** monolayer look darker in the photograph. The peak wavelength of the band-edge absorption was 446.5 nm in the dispersion and 447.4 nm in the monolayer, which were almost the same (Figure 2b­(iv)). In contrast, the peak wavelengths of the band-edge emission were 448.1 and 453.7 nm, and the 6.7 nm increase in the Stokes shift was attributed to monolayer formation. The emission wavelength of a QD strongly depends on its size and can be essentially altered even by a subtle size distribution, resulting in a shift in the wavelength toward the lower energy side through energy transfer between adjacent QDs.^45^ The increase in the Stokes shift compared with the dispersed state is thus attributed to energy transfer in the close-packed 2D superlattice. We succeeded in fabricating uniformly arranged monolayers using spherical Au NPs and CdS QDs, which is a significant step toward the formation of multilayered structures.

### Formation of NP Multilayers

First, a unary multilayer was fabricated using a single type of NP to verify the accuracy of the multilayering process. Figure 3a–c shows the UV/vis extinction spectra of unary multilayers of **A** and **Q** and the PL spectra of **Q** from the monolayer (1L) to 5 layers (5L). In addition, the extinction or PL intensities were plotted as a function of the number of layers (Figure 3d–f). The extinction and PL intensities were proportional to the number of layers, indicating that each monolayer was properly transferred and multilayered on the substrate, in which the extinction at 600.0 nm in Figure 3a corresponds to the LSPR band of **A** and the absorbance at 447.4 nm in Figure 3b corresponds to the band-edge absorption of **Q**. Notably, there was almost no spectral overlap between the LSPR band of **A** and the absorption of **Q** for PL.

**Figure 3:**
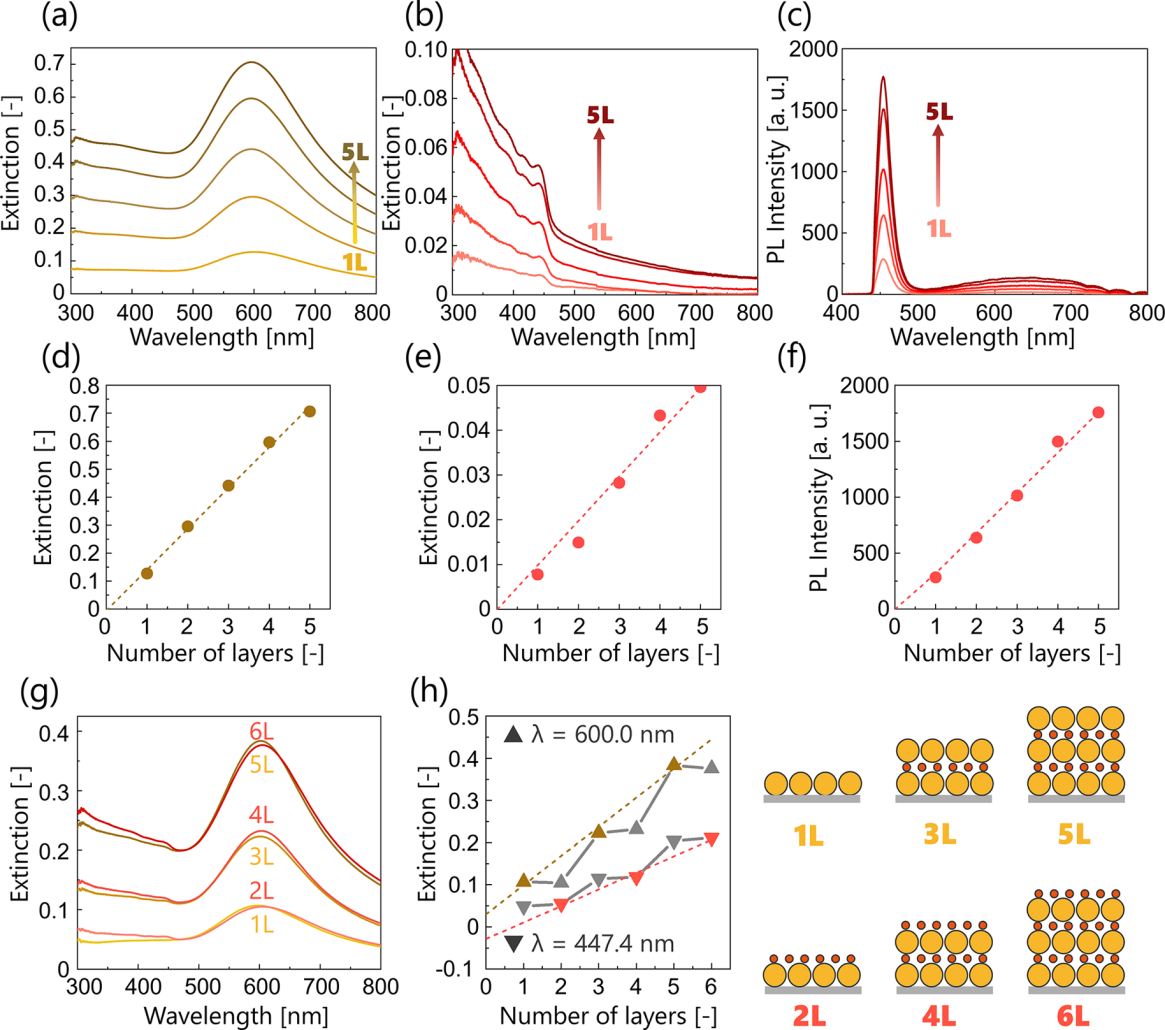
UV/vis extinction spectra of unary multilayers of (a) **A** and (b) **Q**, and (c) PL spectra of **Q** (excitation wavelength: 400 nm) from 1L to 5L. Extinction at wavelengths of (d) 600.0 nm and (e) 447.4 nm and (f) PL intensity at 453.7 nm in (a), (b), and (c) as a function of the number of layers. (g) Extinction spectra of **A**/**Q**. (h) Extinction at wavelengths of 600.0 nm (triangles) and 447.4 nm (inverted triangles) as a function of the number of layers. The ochre dotted line is the line fitted through 1, 3, and 5L at λ = 600.0 nm in the extinction plot, whereas the red line is the line fitted through 2, 4, and 6L at λ = 447.4 nm. The schematic illustrations on the right side represent each multilayer. Yellow sphere: **A**; red sphere: **Q**. All the measurements were not repeated (*n* = 1).

Next, a binary multilayer was fabricated by alternately laminating **A** and **Q** monolayers, with the **A** monolayer as the bottom layer. Here, **A**/**Q** represents the alternating binary multilayers composed of **A** and **Q** layers. **A**/**Q** absorb both types of light at wavelengths of 600.0 and 447.4 nm because of the LSPR band of **A** and the band-edge absorption of **Q** (Figure 3g). Figure 3h shows a linear increase in the extinction at 600.0 and 447.4 nm as the corresponding layer number increases (see the dotted lines in Figure 3h). In these plots, the absorbance at 447.4 nm increased as the number of **A** layers increased due to interband absorption (see gray inverted triangles), whereas that at 600.0 nm did not increase as the number of **Q** layers increased (see gray triangles). In addition to the overlap with the interband absorption of **A**, the absorption of **Q** was subtle owing to the smaller diameter compared with that of **A**, resulting in a smaller increase at 447.4 nm. Moreover, the average value of the amount of increase at 447.4 nm with multilayering was almost identical to the extinction of **Q** 1L. These results verified proper lamination of **A**/**Q**.

### Structural Analysis of Multilayers

To understand the relationship between the spatial arrangement of the NPs and their PL properties, structural analysis was performed. Before we focus on complicated BNSLs, we discuss the structural analysis of the unary multilayers. TEM images of 1L, 2L, and 3L unary multilayers of **A** are shown in Figure 4a–c. The FFT patterns of each TEM image, showing high periodicity of the mono/multilayers, are shown in Figure S3. The laminated order resembled the (111) plane overlap in a face-centered cubic (FCC) structure; the NPs in the second layer were positioned between those in the first layer, whereas the NPs in the third layer occupied sites distinct from those in both the first and second layers. Further structural analysis of the unary multilayer of **A** was conducted using GI-SAXS measurements on 5L. As shown in Figure 4d, the GI-SAXS pattern well matched that of the FCC structure with a lattice constant of 19.6 nm, demonstrating that **A** formed an FCC structure upon lamination of its hexagonally arranged monolayers. The results from the TEM observations and GI-SAXS measurements clearly indicated that the **A** monolayers were laminated similar to (111) planes in the FCC structure (see Figure 4e–g). In principle, spherical NPs coated with alkyl ligands (e.g., oleylamine, oleic acid, and DT) tend to self-assemble into close-packed structures upon the evaporation of organic solvents via thermodynamic interactions between ligands.^11^ Our study thus indicates that close-packed 3D structures can be artificially tailored through the multilayering method.

**Figure 4:**
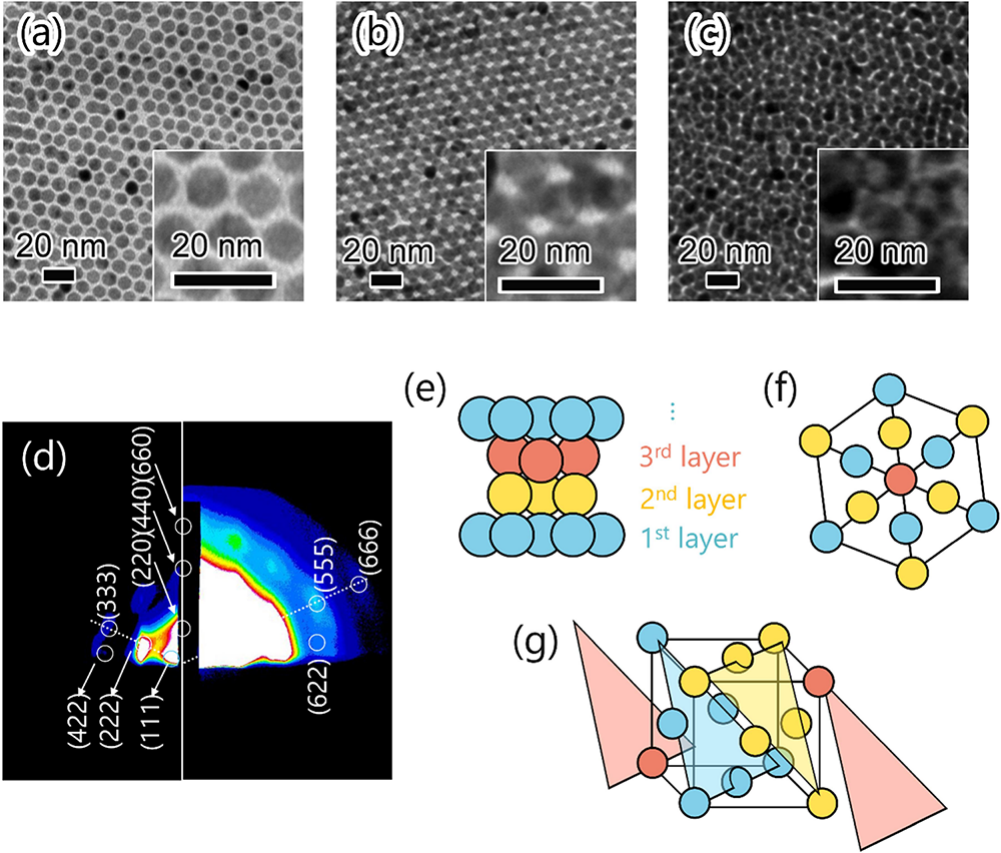
TEM images of (a) 1L, (b) 2L, and (c) 3L **A**. (d) GI-SAXS pattern of **A** 5L. (e–g) Schematics of the FCC structure formed by stacking of **A** monolayers: (e) perpendicular view to the stacking direction, (f) (111) view, and (g) oblique view of a unit cell.

The structural analysis of the alternating binary multilayer, **A**/**Q**, involves evaluating the impact of inserting **Q** layers on the structure of the unary multilayer of **A**. Figure 5a presents the GI-SAXS pattern obtained for **A**/**Q** 5L. Several spot-like scatterings, which indicate the formation of a long-range ordered regular arrangement, appeared in the pattern for **A**/**Q**, and the corresponding lattice spacings were in good agreement with those of an FCC-type structure as well as **A** 5L. Even though GI-SAXS measurements were performed on **A**/**Q** 6L, significant patterns as clear as those for 5L could not be obtained, likely due to reduced structural symmetry. As a related example, we also observed that the scattering pattern of unary multilayers varied depending on whether the number of layers was odd or even. The lattice constant of **A**/**Q** increased to 20.8 nm upon alternating lamination of **A** and **Q** monolayers, suggesting the formation of a structure distinct from the FCC structure. Additionally, the interlayer distance between **A** layers, which is identical to the (111) plane spacing, expanded to 12.0 nm in the binary multilayer compared to the value of 11.3 nm in the unary multilayer of **A**. The increase in the plane spacing for **A**/**Q** was less than the thickness of the **Q** layer (3.3 nm in diameter). This observation suggests that the **Q** layer was deposited onto gaps between **A** particles in the lower layer, resulting in the formation of a BNSL in which the two lattices were effectively integrated.

**Figure 5:**
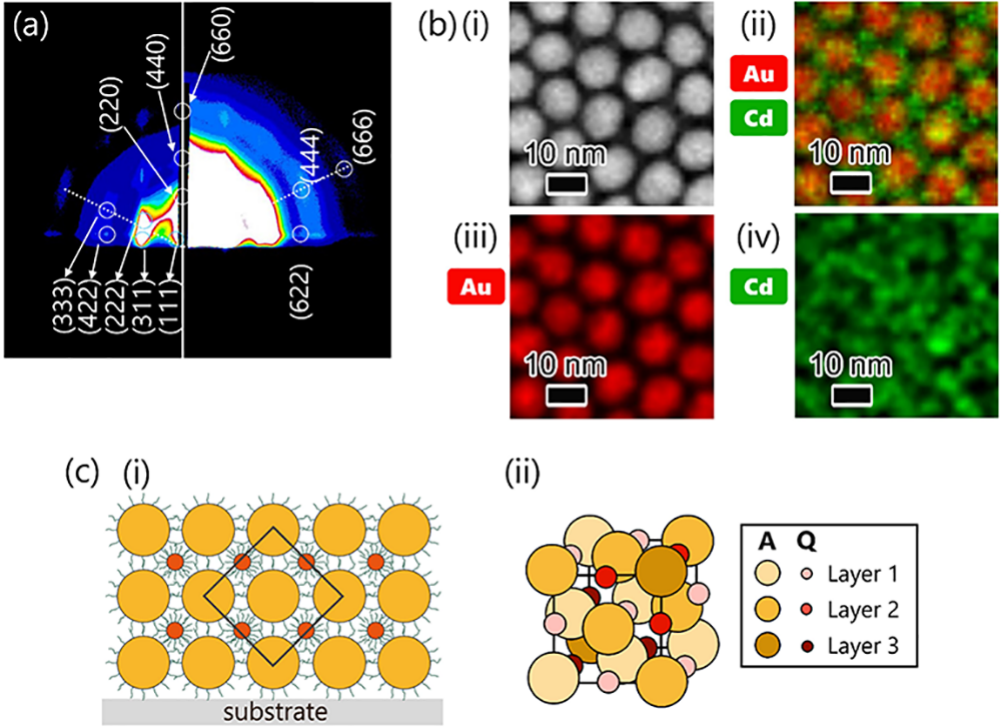
(a) GI-SAXS pattern of **A**/**Q** 5L. (b) (i) HAADF-STEM image and (ii-iv) EDS images of **A**/**Q** 2L. EDS elemental mapping was performed for Au and Cd, and each image shows the results of (ii) an overlay of Au and Cd, (iii) Au and (iv) Cd. (c) Schematics of the estimated multilayer structure of **A**/**Q**. (i) Side view with a substrate. The black frame corresponds to the perimeter of the unit cell illustrated in (ii). The color of the NP spheres was set according to the layer number.

Notably, almost all spot-like scattering patterns originated from **A**, as the scattering intensities from **Q** were too weak to acquire meaningful information (Figure S4). High-angle annular dark field (HAADF)-STEM observations and energy-dispersive X-ray spectroscopy (EDS) mapping were thus conducted on **A**/**Q** 2L to visualize the stacking manner along the [111] direction. According to the HAADF image (Figure 5b­(i)) and EDS mapping results (Figure 5b­(ii–iv)), six **Q**s surrounded one **A** in the lower layer, and one **Q** was on the **A**, corresponding to the (111) plane of a NaCl-type structure, whereas there was the possibility of the formation of other FCC-type structures, such as zinc blends, if the **Q** layer was only above the **A** layer. Here, the NaCl-type structure, which is the overlap of two FCC arrays, could be one option for the structure of **A**/**Q**. This assumption can be supported by the observation that the increase in the interlayer distance caused by inserting a **Q** layer was less than the thickness of the **Q** layer. However, the arrangement of **Q** in the FCC array formed by **A** has not yet been determined. The detailed structure might be clarified by measuring anomalous scattering from cadmium and predicting an ideal scattering pattern using calculations.

Figure 5c shows the estimated multilayer structure of **A**/**Q** drawn from the results and discussion thus far. For most cases of BNSL formation facilitated by solvent evaporation, competitive Coulomb interactions between ligands in the solvent, steric repulsion, and van der Waals attraction are major driving forces that stabilize the BNSL structure.^26,31,32,46^ In our case, the NP layers were artificially laminated in an almost dry state, in which interactions between ligands at the interface of adjacent layers likely guided the NPs to their most stable positions. Entropy, which works to stabilize the van der Waals attraction between metal and semiconductor cores and the repulsion between hydrophobic ligands, thus appears to play a significant role as a dominant driving force in the formation of the 3D structure during the multilayering process, rather than electrostatic interactions.

In BNSL formation through lamination of two types of NP monolayers, the NP arrangement could be optimized through interaction and size matching between the different cores transferred from an air/water interface with a planar surface to a different NP layer with an uneven surface. In the end, we succeeded in creating a BNSL system composed of Au NPs and QDs functionalized with alkanethiol ligands through accurate multilayering.

### Photoluminescence Properties of Alternating Binary Multilayers

The photoelectric field generated by the LSPR of the Au NPs influences the photoexcitation and emission processes of the QDs, thereby affecting the overall emission intensity. As shown in Figure 3, the LSPR absorption wavelength of **A** (∼600 nm) does not overlap with the excitation wavelength of **Q** at 400 nm, ensuring that no excitation enhancement occurs under the experimental conditions. The degree of PL enhancement or quenching depends on the overlap between the emission and LSPR absorption wavelengths, as well as the distance between the QDs and Au NPs.^47–51^ In the present system, minimal spectral overlap is observed between the emission spectrum of **Q** 1L and the extinction spectrum of **A** 1L (Figure S5), suggesting that distance-dependent enhancement and quenching effects are negligible.

The resulting PL spectra of **A**/**Q** with multilayering are shown in Figure 6a. The above interpretation is supported by the nearly identical band-edge emission intensities for **Q** 1L and **A**/**Q** 2L (Figure 6b), indicating that plasmon–exciton interactions do not significantly affect the emission, despite the close proximity of the NP layers confirmed by structural analysis. Notably, PL of **A**/**Q** was never quenched even when **A** layer was laminated, indicating the potential for step-by-step PL tuning. In contrast, the PL intensity in **A**/**Q** 3L is significantly lower than that in **A**/**Q** 2L, and this tendency appears for the **A**/**Q** multilayers with an odd number of layers (Figure 6b). This change in the PL intensity was confirmed by the naked eye via fluorescence microscopy images (Figure S6).

**Figure 6:**
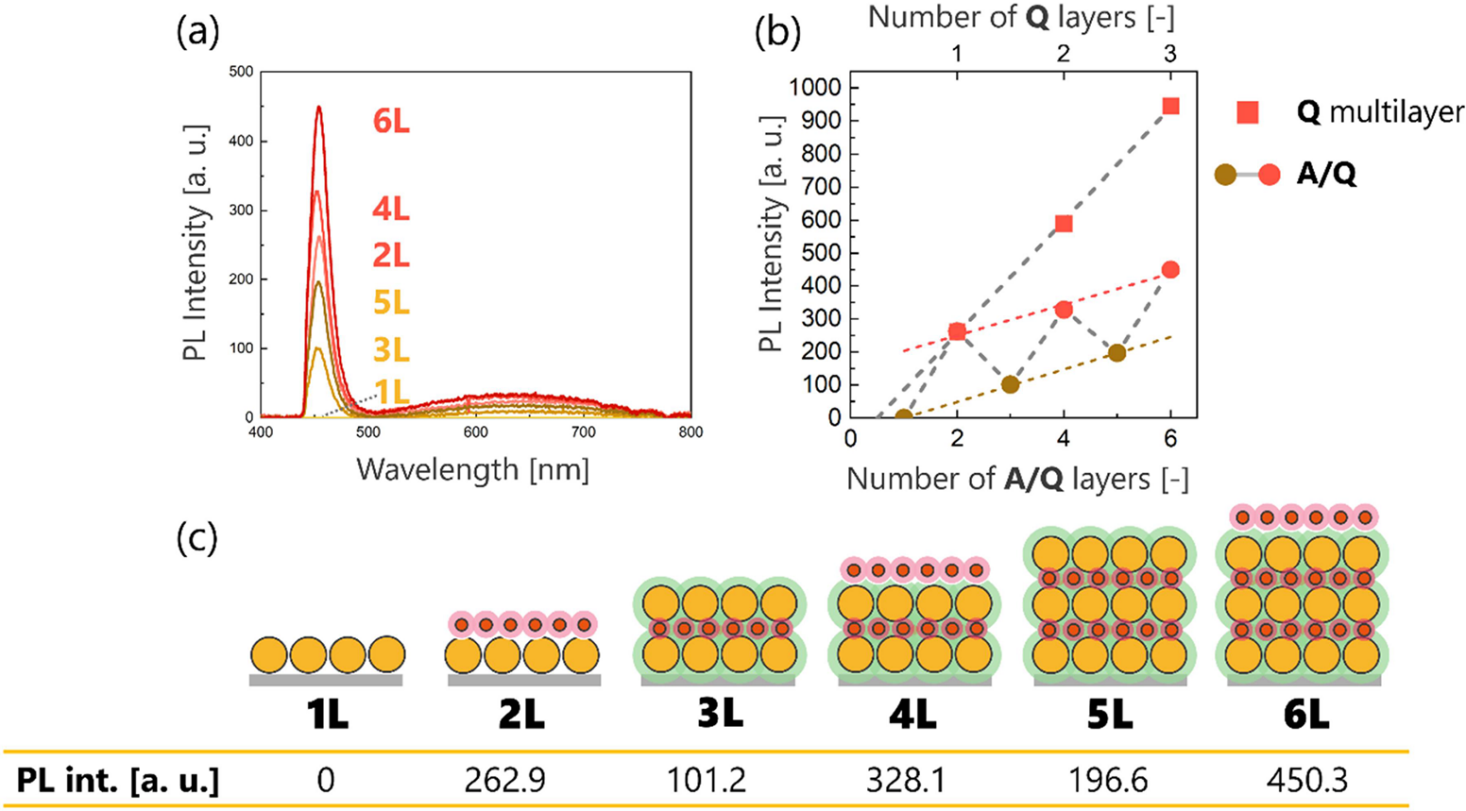
(a) PL spectra of **A**/**Q** multilayers from 1L to 5L. (b) PL intensities at a wavelength of 453.7 nm in (a) and in Figure 3c as a function of the number of layers (*n* = 1). As a baseline correction, the PL intensity of **A**/**Q** 1L was subtracted from the other **A**/**Q** data. The ochre dotted line is the line fitted through the odd-number **A**/**Q** layers, whereas the red line is the line fitted through the even-number **A**/**Q** layers. (c) Schematic illustrations of **A**/**Q** multilayers, where the green and pink regions correspond to the **AQA** layer and the **Q** adlayer, respectively. The absolute values of the PL peak intensities are presented in the table.

The reason for this behavior is not the interaction between excitons and plasmons but the optical configuration described below. In our PL measurements, both irradiation with excitation light and detection of light emitted from the sample were performed from the top surface of the multilayer film under a fluorescence microscope. From the perspective of this measurement system, an **A** layer could act as an optical attenuator for emission from a **Q** layer. If the optical absorbance at the emission wavelength of the **A** layer is denoted as *A*λ, then the relationship between the transmitted light intensity (*I*_*n*+1_) and incident light intensity (*I_n_*) can be described as *A*λ = −log10­(*I*_*n*+1_/*I_n_*), where *n* is the number of layers and *I*_*n*+1_/*I_n_* is the ratio of the optical attenuation by the **A** adlayer. Based on the PL intensity data, *A*λ was estimated to be 0.415 from the value of *I*_3_/*I*_2_ = 0.385. However, the absorbance of **A** 1L at the band-edge emission wavelength was only 0.0492, indicating that the optical attenuation could not be explained only by the absorbance of the **A** adlayer. Additionally, since the PL intensity was clearly linearly correlated with both the number of even layers and the number of odd layers, with higher and lower values, respectively, an optical phenomenon originating from the periodic stacking structure was suggested (Figure 6b).

We carried out FDTD simulations using an **A**/**Q** layer model in which **A** and **Q** layers were assumed to form a simple laminated structure as two independent monolayers (Figure S7). The simulation results revealed a similar trend in the PL intensity change with multilayering, but the optical attenuation ratio was constant, instead of the optical attenuation value, which was not consistent with the experimental results.

We therefore propose a new model with an “**AQA** layer” (marked by the green background in Figure 6c), in which a **Q** layer is sandwiched by **A** layers as a layer with a homogeneous medium. A **Q** adlayer (marked by a pink background in Figure 6c) was placed on this **AQA** layer. The average PL intensities of the **AQA** layer and the **Q** layer were estimated to be 99.8 ± 1.39 and 247.3 ± 5.85 (a.u.), respectively. For example, the PL intensity of **A**/**Q** 4L seemed to agree with their sum, as shown in the table in Figure 6c, although there were some errors.

FDTD simulation was performed to verify the validity of the hypothesis. In the **A**/**Q** 4L model (Figure 7a­(i)), an emitting layer (plane light source) was placed in the middle of the **AQA** layer (23 nm thickness) and the **Q** adlayer (3 nm thickness). Here, the optical parameters of the **AQA** layer were assumed to be identical to those of the **A** layer (the derivation of its parameters is described in the Experimental section) since the influence of the **Q** layer was negligible (see the extinction spectra of **A**/**Q** in Figure 3g). The repeat structure of **AQA** (e.g., **AQAQA**) was assumed to be an analog of **AQA**, as schematically depicted in Figure 7a­(ii) for **A**/**Q** 5L. The PL intensity simulated with this layered model was in good agreement with the experimental data (Figure 7b,c), in which the decrease in the PL intensity from EVEN to ODD layer numbers (*I*_*n*+1_ – *I_n_*) was constant but the attenuation ratio (*I*_*n*+1_/*I_n_*) was not (the original simulation data are available in Figure S8).

**Figure 7:**
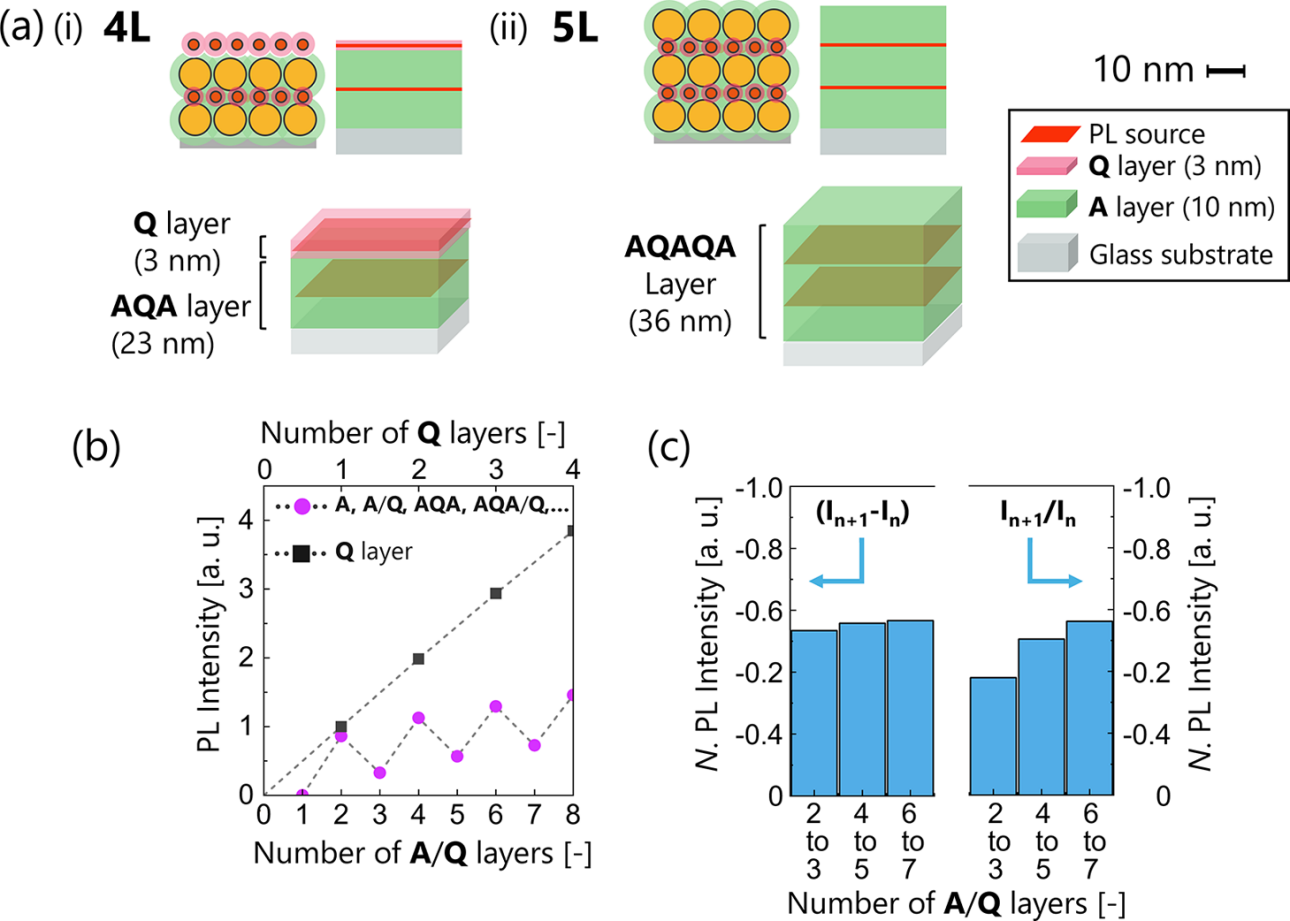
(a) Schematics of **A**/**Q** (i) 4L and (ii) 5L for FDTD simulation. Here, **A**/**Q** 4L was described as a combination of an **AQA** layer and a **Q** adlayer. **A**/**Q** 5L was described as an **AQAQA** layer. (b) FDTD simulation results of the PL intensity as a function of the number of layers, assuming the formation of an **AQA** layer. (c) Decrease in the PL intensity (three bars on the left) or attenuation ratio (right) relative to the lower layer. The decrease amount was constant, which agreed with the experimental results.

The BNSL structure of the **AQA** repeat unit, formed through alternate multilayering, facilitated the creation of an exceptional optical environment distinct from that of the **Q** adlayer at the top surface of the multilayers. This unique configuration, which cannot be achieved through simple mixing, offers diverse ways to tune the optical properties. Furthermore, the fact that films prepared by such a simple fabrication method, i.e., stacking NP monolayers, have structural and optical characteristics similar to those of BNSL materials with well-defined multilayered structures suggests that this technique has the potential to create novel optical metamaterials using NPs as building blocks. In particular, the optical properties of multilayered films can be tuned by adjusting the number of layers, which is a major advantage for rationally designing optical metamaterials.

## Conclusions

In this work, a binary multilayered structure composed of DT-modified Au NPs and CdS QDs was successfully fabricated through alternate lamination of their hexagonally arranged monolayers spread on a water surface. GI-SAXS measurements confirmed the high periodicity of the binary alternating multilayer, with the Au NPs forming an FCC structure. Notably, the increase in the interlayer distance resulting from the insertion of the QD layer, as determined by GI-SAXS results, was less than the estimated QD layer thickness. Furthermore, STEM-EDS observations revealed that CdS QDs were deposited around Au NPs. These results indicated the formation of a BNSL in which the lattices of Au NPs and CdS QDs were merged. The systematic change in the PL intensity caused by alternate multilayering was due to BNSL formation rather than by a simple laminated structure of two independent layers and was in good agreement with the results of FDTD simulations assuming no dielectric function interfaces within the multilayered structure.

The method proposed in this study allows the production of ultrathin BNSLs with controllable thickness at the level of a single NP layer on a macroscopic surface under ambient conditions. Furthermore, the fabricated BNSL films exhibit not only precisely defined multilayered structures but also optical properties that systematically vary with the number of layers. Thus, this multilayer BNSL fabrication method has great potential for industrial applications and offers significant advantages over traditional BNSL fabrication methods that rely on solvent evaporation under reduced pressure and moderately high-temperature conditions. Moreover, if plasmon–exciton coupling can be effectively achieved by further optimizing the LSPR band position via the NP size and self-assembled structure, the application potential of these ultrathin BNSL films should be further enhanced. NP arrays with different structures and functions can be created by using NPs modified with different surface ligands, based on the present multilayer system as a model structure. It will be a versatile platform for creating novel optical metamaterials with tailored functionalities. Further studies using dendritic ligands are currently in progress.

## Supplementary Material



## Notes

Photograph, TEM image, FFT pattern, and UV/vis extinction spectra of Au NP monolayers with chloroform as the solvent; FFT patterns of 1–3L of **A**; GI-SAXS pattern of 2L of **Q**; emission and the extinction spectra of monolayers; fluorescence microscopy images of **A**/**Q** and **Q**; and FDTD simulation results (PDF)

## References

[1] Guo J., Guo S., Su X., Zhu S., Pang Y., Luo W., Zhang J., Sun H., Li H., Zhang D. (2020). Nonvolatile
Resistive Switching Memory Device Employing CdSe/CdS Core/Shell Quantum
Dots as an Electrode Modification Layer. ACS
Appl. Electron. Mater..

[2] Kim G. Y., Kim S., Choi J., Kim M., Lim H., Nam T. W., Choi W., Cho E. N., Han H. J., Lee C., Kim J. C., Jeong H. Y., Choi S. Y., Jang M. S., Jeon D. Y., Jung Y. S. (2019). Order-of-Magnitude,
Broadband-Enhanced
Light Emission from Quantum Dots Assembled in Multiscale Phase-Separated
Block Copolymers. Nano Lett..

[3] Jiang K., Smith D. A., Pinchuk A. (2013). Size-Dependent
Photothermal Conversion
Efficiencies of Plasmonically Heated Gold Nanoparticles. J. Phys. Chem. C.

[4] Kanie K., Matsubara M., Zeng X., Liu F., Ungar G., Nakamura H., Muramatsu A. (2012). Simple Cubic Packing of Gold Nanoparticles
through Rational Design. J. Am. Chem. Soc..

[5] Matsubara M., Stevenson W., Yabuki J., Zeng X., Dong H., Kojima K., Chichibu S. F., Tamada K., Muramatsu A., Ungar G., Kanie K. (2017). A Low-Symmetry Cubic Mesophase of
Dendronized CdS Nanoparticles and Their Structure-Dependent Photoluminescence. Chem..

[6] Han X., Liu Y., Yin Y. (2014). Colorimetric Stress Memory Sensor
Based on Disassembly
of Gold Nanoparticle Chains. Nano Lett..

[7] Cha H., Hee Yoon J., Yoon S. (2014). Probing Quantum
Plasmon Coupling
Using Gold Nanoparticle Dimers with Tunable Interparticle Distances
Down to the Subnanometer Range. ACS Nano.

[8] Kodaimati M. S., Wang C., Chapman C., Schatz G. C., Weiss E. A. (2017). Distance-Dependence
of Interparticle Energy Transfer in the Near-Infrared within Electrostatic
Assemblies of PbS Quantum Dots. ACS Nano.

[9] Nakazawa N., Zhang Y., Liu F., Ding C., Hori K., Toyoda T., Yao Y., Zhou Y., Hayase S., Wang R., Zou Z., Shen Q. (2019). The Interparticle Distance
Limit for Multiple Exciton Dissociation in PbS Quantum Dot Solid Films. Nanoscale Horiz..

[10] Xu J., Shang M., Ni X., Cao Y. (2020). Strategy Based on Rapid
Self-Assembly of Magnetic Nanoparticles for Construction of Photonic
Crystals. ACS Appl. Nano. Mater..

[11] Mohapatra J., Elkins J., Xing M., Guragain D., Mishra S. R., Liu J. P. (2021). Magnetic-Field-Induced
Self-Assembly of FeCo/CoFe2O4core/Shell
Nanoparticles with Tunable Collective Magnetic Properties. Nanoscale.

[12] Schulz F., Pavelka O., Lehmkühler F., Westermeier F., Okamura Y., Mueller N. S., Reich S., Lange H. (2020). Structural
Order in Plasmonic Superlattices. Nat. Commun..

[13] Santos P. J., Macfarlane R. J. (2020). Reinforcing
Supramolecular Bonding with Magnetic Dipole
Interactions to Assemble Dynamic Nanoparticle Superlattices. J. Am. Chem. Soc..

[14] Gao T., Yachi T., Shi X., Sato R., Sato C., Yonamine Y., Kanie K., Misawa H., Ijiro K., Mitomo H. (2024). Ultrasensitive Surface-Enhanced
Raman Scattering Platform
for Protein Detection via Active Delivery to Nanogaps as a Hotspot. ACS Nano.

[15] Nakamura S., Mitomo H., Suzuki S., Torii Y., Sekizawa Y., Yonamine Y., Ijiro K. (2022). Self-Assembly
of Gold Nanorods into
a Highly Ordered Sheet via Electrostatic Interactions with Double-Stranded
DNA. Chem. Lett..

[16] Shen C., Matsubara M., Yabushita M., Maki S., Muramatsu A., Kanie K. (2020). Magnetic Field Induced Uniaxial Alignment of the Lyotropic Liquid-Crystalline
PMMA-Grafted Fe3O4 Nanoplates with Controllable Interparticle Interaction. Nanoscale Adv..

[17] Ross M. B., Blaber M. G., Schatz G. C. (2014). Using Nanoscale
and Mesoscale Anisotropy
to Engineer the Optical Response of Three-Dimensional Plasmonic Metamaterials. Nat. Commun..

[18] Mitomo H., Takeuchi C., Sugiyama R., Tamada K., Ijiro K. (2022). Thermo-Responsive
Silver Nanocube Assembled Films. Bull. Chem.
Soc. Jpn..

[19] Kinjo S., Baliunaite E., Kajino Y., Aida Y., Arima Y., Okamoto K., Tamada K. (2024). Two-Dimensional Empty Liquid Composed
of a Patchy Metal Nanoparticle Network Structure for Transparent Plasmonic
Devices. ACS Appl. Nano. Mater..

[20] Wang K., Ling H., Bao Y., Yang M., Yang Y., Hussain M., Wang H., Zhang L., Xie L., Yi M., Huang W., Xie X., Zhu J. (2018). A Centimeter-Scale
Inorganic Nanoparticle Superlattice Monolayer with Non-Close-Packing
and Its High Performance in Memory Devices. Adv. Mater..

[21] Lambert K., Čapek R. K., Bodnarchuk M. I., Kovalenko M. V., Van Thourhout D., Heiss W., Hens Z. (2010). Langmuir–Schaefer
Deposition of Quantum Dot Multilayers. Langmuir.

[22] Yoshida A., Imazu K., Li X., Okamoto K., Tamada K. (2012). Spectroscopic
Properties of Multilayered Gold Nanoparticle 2D Sheets. Langmuir.

[23] Dong A., Ye X., Chen J., Murray C. B. (2011). Two-Dimensional Binary and Ternary
Nanocrystal Superlattices: The Case of Monolayers and Bilayers. Nano Lett..

[24] Evers W. H., Friedrich H., Filion L., Dijkstra M., Vanmaekelbergh D. (2009). Observation
of a Ternary Nanocrystal Superlattice and Its Structural Characterization
by Electron Tomography. Angew. Chem., Int. Ed..

[25] Paik T., Diroll B. T., Kagan C. R., Murray C. B. (2015). Binary and Ternary
Superlattices Self-Assembled from Colloidal Nanodisks and Nanorods. J. Am. Chem. Soc..

[26] Shevchenko E. V., Talapin D. V., Kotov N. A., O’Brien S., Murray C. B. (2006). Structural Diversity in Binary Nanoparticle
Superlattices. Nature.

[27] Cargnello M., Diroll B. T., Gaulding E. A., Murray C. B. (2014). Enhanced
Energy
Transfer in Quasi-Quaternary Nanocrystal Superlattices. Adv. Mater..

[28] Dong A., Chen J., Vora P. M., Kikkawa J. M., Murray C. B. (2010). Binary
Nanocrystal Superlattice Membranes Self-Assembled at the Liquid-Air
Interface. Nature.

[29] Josten E., Wetterskog E., Glavic A., Boesecke P., Feoktystov A., Brauweiler-Reuters E., Rücker U., Salazar-Alvarez G., Brückel T., Bergström L. (2017). Superlattice Growth and Rearrangement
during Evaporation-Induced Nanoparticle Self-Assembly. Sci. Rep..

[30] Talapin D. V., Shevchenko E. V., Bodnarchuk M. I., Ye X., Chen J., Murray C. B. (2009). Quasicrystalline
Order in Self-Assembled Binary Nanoparticle
Superlattices. Nature.

[31] Chen Z., O’Brien S. (2008). Structure
Direction of II-VI Semiconductor Quantum
Dot Binary Nanoparticle Superlattices by Tuning Radius Ratio. ACS Nano.

[32] Shevchenko E. V., Talapin D. V., Murray C. B., O’Brien S. (2006). Structural
Characterization of Self-Assembled Multifunctional Binary Nanoparticle
Superlattices. J. Am. Chem. Soc..

[33] Smith D. K., Goodfellow B., Smilgies D. M., Korgel B. A. (2009). Self-Assembled Simple
Hexagonal AB2 Binary Nanocrystal Superlattices: SEM, GISAXS, and Defects. J. Am. Chem. Soc..

[34] Oliveira O. N., Caseli L., Ariga K. (2022). The Past and
the Future of Langmuir
and Langmuir-Blodgett Films. Chem. Rev..

[35] Zheng N., Fan J., Stucky G. D. (2006). One-Step One-Phase Synthesis of Monodisperse Noble-Metallic
Nanoparticles and Their Colloidal Crystals. J. Am. Chem. Soc..

[36] Liu X., Jiang Y., Lan X., Li S., Wu D., Han T., Zhong H., Zhang Z. (2011). Synthesis of High Quality and Stability
CdS Quantum Dots with Overlapped Nucleation-Growth Process in Large
Scale. J. Colloid Interface Sci..

[37] Bethune D. S. (1989). Optical
Harmonic Generation and Mixing in Multilayer Media: Analysis Using
Optical Transfer Matrix Techniques. J. Opt.
Soc. Am. B.

[38] Okamoto K., Tanaka D., Degawa R., Li X., Wang P., Ryuzaki S., Tamada K. (2016). Electromagnetically Induced Transparency
of a Plasmonic Metamaterial Light Absorber Based on Multilayered Metallic
Nanoparticle Sheets. Sci. Rep..

[39] Treharne R. E., Seymour-Pierce A., Durose K., Hutchings K., Roncallo S., Lane D. (2011). Optical Design
and Fabrication of
Fully Sputtered CdTe/CdS Solar Cells. J. Phys.:
Conf. Ser..

[40] Swierczewski M., Bürgi T. (2023). Langmuir and Langmuir-Blodgett Films of Gold and Silver
Nanoparticles. Langmuir.

[41] Huang S., Tsutsui G., Sakaue H., Shingubara S., Takahagi T. (2001). Experimental Conditions for a Highly
Ordered Monolayer
of Gold Nanoparticles Fabricated by the Langmuir–Blodgett Method. J. Vac. Sci. & Technol. B.

[42] Huang S., Tsutsui G., Sakaue H., Shingubara S., Takahagi T. (2001). Formation of a Large-Scale Langmuir-Blodgett
Monolayer
of Alkanethiol-Encapsulated Gold Particles. J. Vac. Sci. Technol. B.

[43] Pei L., Mori K., Adachi M. (2006). Investigation on Arrangement and
Fusion Behaviors of Gold Nanoparticles at the Air/Water Interface. Colloids Surf. A-Physicochem. Eng. Asp..

[44] Tamura M., Okamoto K., Tamada K., Iida T. (2018). Stochastic Approach
to Simulation of Evaporation-Triggered Multiple Self-Assembly of Mixed
Metal Nanoparticles and Their Variable Superradiance. Appl. Phys. Lett..

[45] Michalet X., Pinaud F. F., Bentolila L. A., Tsay J. M., Doose S., Li J. J., Sundaresan G., Wu A. M., Gambhir S. S., Weiss S. (2005). Quantum Dots for Live Cells, in Vivo Imaging, and Diagnostics. Science.

[46] Ji N., Chen Y., Gong P., Cao K., Peng D. L. (2015). Investigation
on the Self-Assembly of Gold Nanoparticles into Bidisperse Nanoparticle
Superlattices. Colloids Surf. A Physicochem
Eng. Asp.

[47] Samanta A., Zhou Y., Zou S., Yan H., Liu Y. (2014). Fluorescence
Quenching of Quantum Dots by Gold Nanoparticles: A Potential Long
Range Spectroscopic Ruler. Nano Lett..

[48] Sun D., Tian Y., Zhang Y., Xu Z., Sfeir M. Y., Cotlet M., Gang O. (2015). Light-Harvesting Nanoparticle
Core-Shell
Clusters with Controllable Optical Output. ACS
Nano.

[49] Ribeiro T., Prazeres T. J. V., Moffitt M., Farinha J. P. S. (2013). Enhanced Photoluminescence
from Micellar Assemblies of Cadmium Sulfide Quantum Dots and Gold
Nanoparticles. J. Phys. Chem. C.

[50] Nasrin F., Chowdhury A. D., Takemura K., Kozaki I., Honda H., Adegoke O., Park E. Y. (2020). Fluorometric Virus Detection Platform
Using Quantum Dots-Gold Nanocomposites Optimizing the Linker Length
Variation. Anal. Chim. Acta.

[51] Tobias A. K., Jones M. (2019). Metal-Enhanced Fluorescence
from Quantum Dot-Coupled Gold Nanoparticles. J. Phys. Chem. C.

