# Supplementary material for: Alternating Binary Multilayers of Alkanethiol-Modified Gold Nanoparticles and Quantum Dots with Artificial Three-Dimensional Structures and Rational Photoluminescence
Source: ACS Appl Mater Interfaces. 2025 May 2;17(23):34360–9. doi: 10.1021/acsami.5c02956 (PMC12163927; doi:10.1021/acsami.5c02956)
Supplement: Supplementary file 1 [file am5c02956_si_001.pdf]

Supporting Information

Alternating Binary Multilayers of Alkanethiol-Modified Gold Nanoparticles and Quantum Dots with Artificial Three-Dimensional Structures and Rational Photoluminescence

Rina Sato,[1] Hideyuki Mitomo,[2] Yuto Kajino,[3] Masaki Matsubara,[4,5] Takehiro Yachi,[1] Megumi Suyama,[1] Kaoru Tamada,[3] and Kiyoshi Kanie*.[1,4]

1: Institute of Multidisciplinary Research for Advanced Materials, Tohoku University, 2-1-1 Katahira, Aoba-ku, Sendai, Miyagi, 980-8577, Japan.

2: Research Institute for Electronic Science, Hokkaido University, Kita 21, Nishi 10, Kita-Ku, Sapporo, Hokkaido, 001-0021, Japan.

3: Institute for Materials Chemistry and Engineering, Kyushu University, 744 Motooka, Nishi-Ku, Fukuoka, 819-0935, Japan.

4: International Center for Synchrotron Radiation Innovation Smart, Tohoku University, 2-1-1 Katahira, Aoba-ku, Sendai, Miyagi, 980-8577, Japan.

5: National Institute of Technology, Sendai College, Nodayama-Shiote 48, Medeshima, Natori, Sendai, Miyagi, 981-1239, Japan.

*Corresponding Author, email: kanie@tohoku.ac.jp

Supplementary Figures

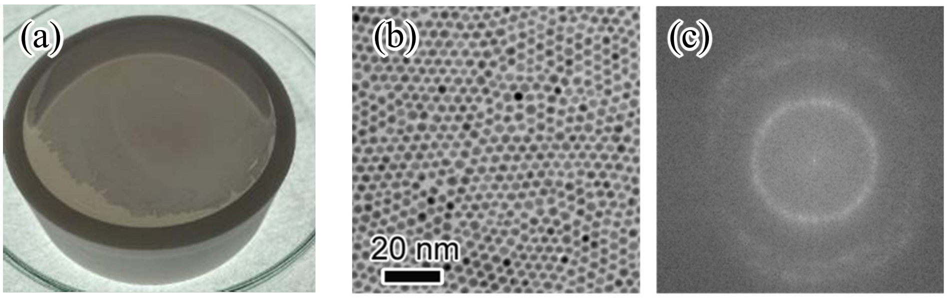

Figure S1 Observation results of Au NP monolayer formed with chloroform as the solvent. (a) A photograph of monolayer on a water surface, (b) TEM image and (c) FFT pattern. Random arranged monolayer was obtained compared with the monolayer using toluene, which results in ring-like FFT pattern.

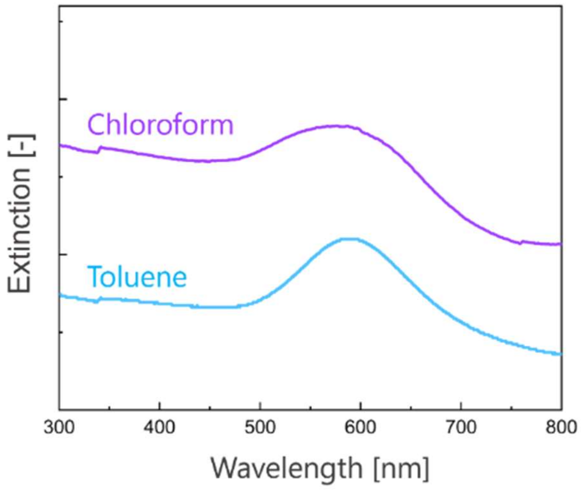

Figure S2 UV/vis extinction spectra of Au NP monolayer formed with chloroform (purple) or toluene (blue) as the solvents.

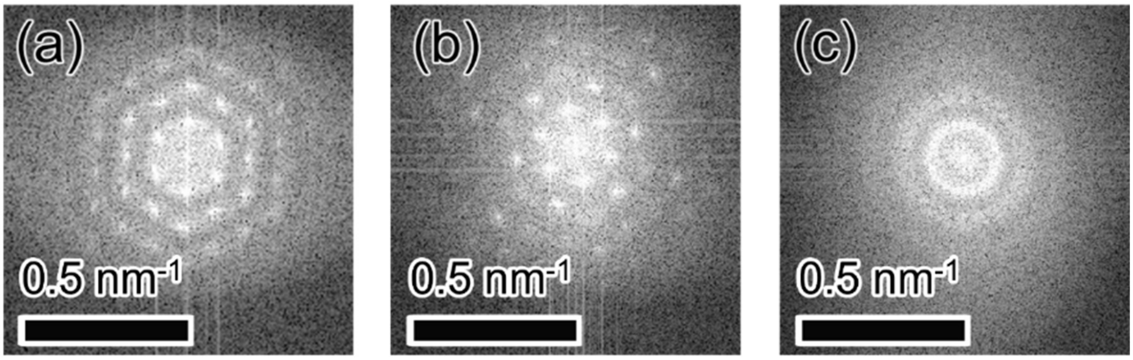

Figure S3 FFT patterns of (a) 1L, (b) 2L, and (c) 3L of **A**.

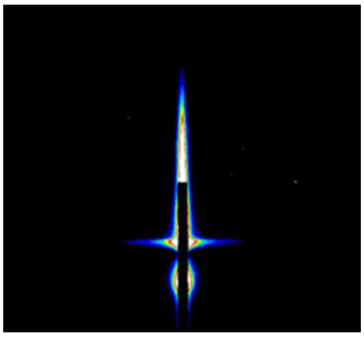

Figure S4 A GI-SAXS pattern of 2L of **Q**.

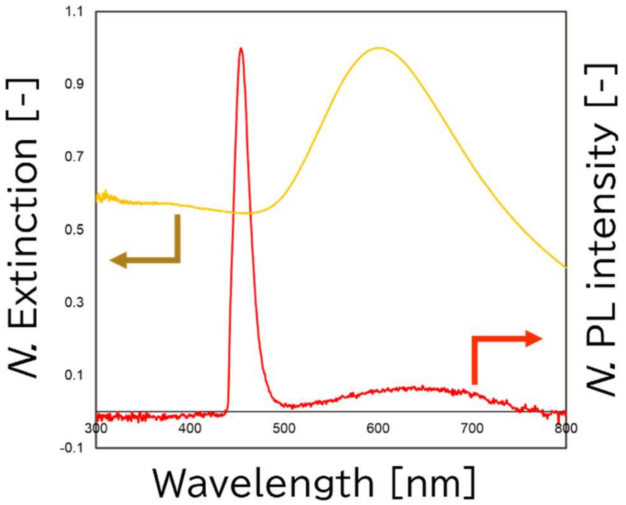

Figure S5 The emission spectrum of 1L of **Q** (red) and the extinction spectrum of 1L of **A** (yellow).

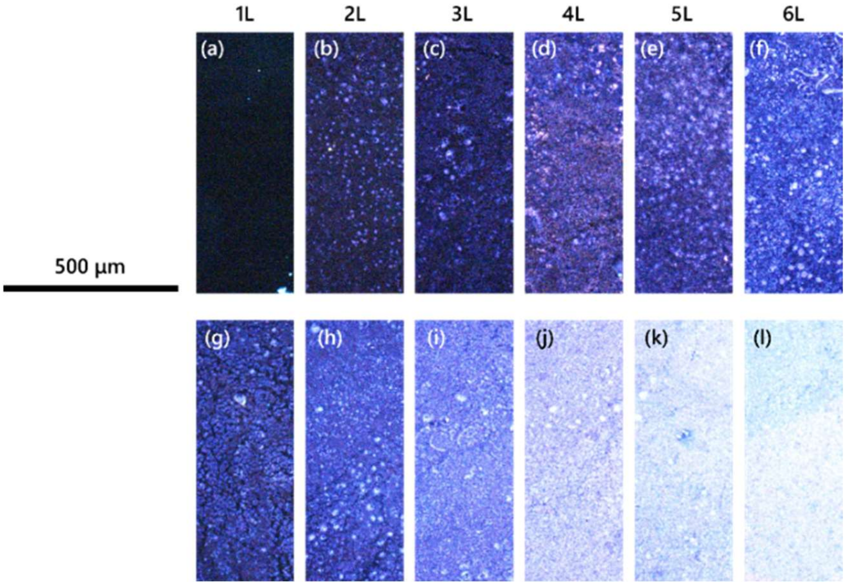

Figure S6 Fluorescence microscopy images of (a-f) **A/Q** and (g-l) **Q** multilayers as laminating. The scale bar is common to all images.

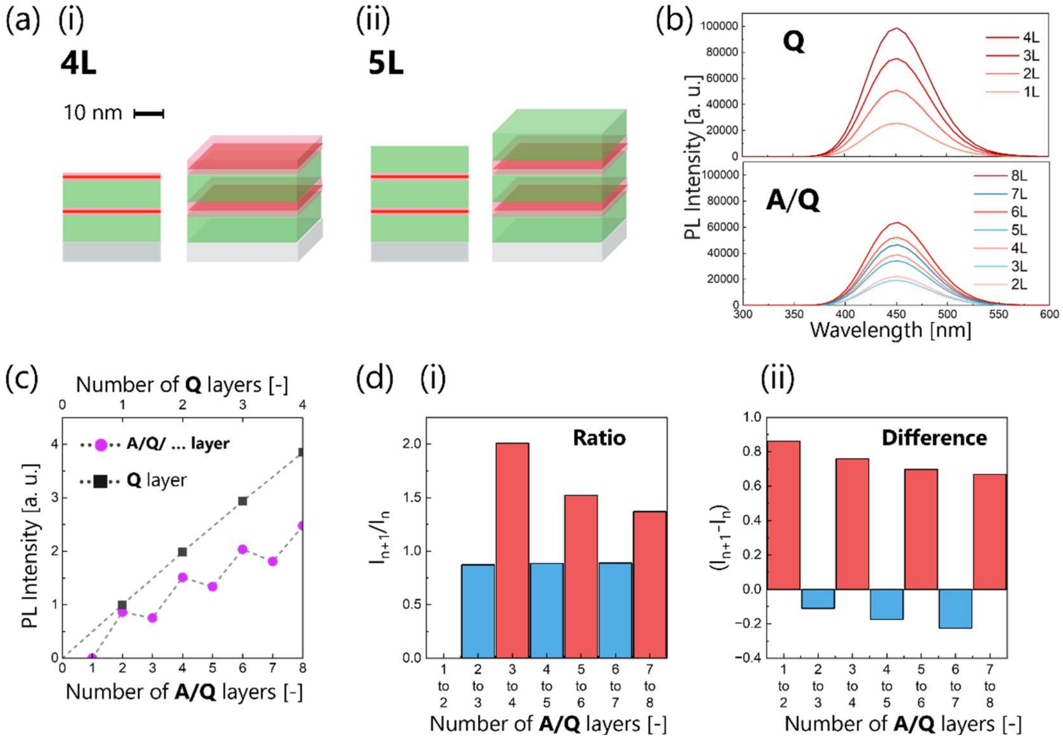

Figure S7 (a) Schematics of **A**/**Q** (i) 4L and (ii) 5L for FDTD simulation using an **A**/**Q** layer model in which **A** and **Q** layers were assumed to form a simple laminated structure as two independent monolayers. Information as to each color in illustration is the same as in **Fig. 9**. (b) FDTD simulation results of the PL spectra of **Q** unary multilayer (top) and **A**/**Q** (bottom). (c) PL intensities in (b) as a function of the number of layers. (d) (i) The ratio and (ii) the difference of PL intensity relative to the lower layer. Red and blue bar represent the increase and the decrease in PL intensity from the lower layer, respectively. PL intensities in (c)(d) were normalized by the simulated PL intensity of 1L of **Q**.

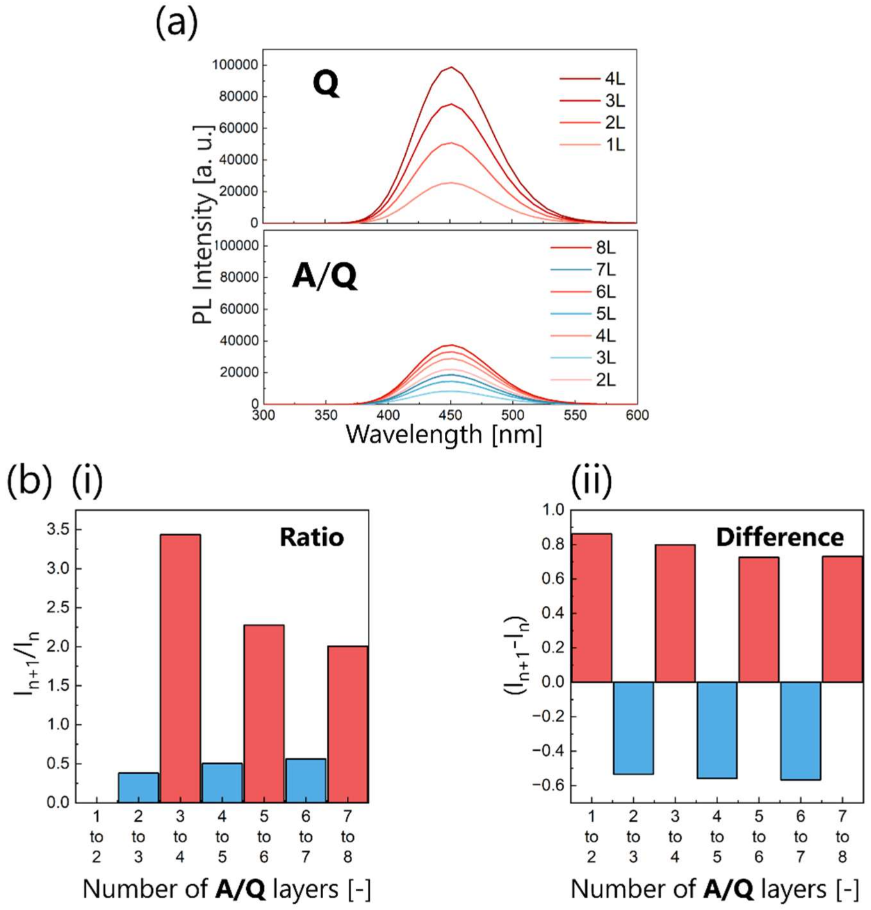

Figure S8 FDTD simulation results using an **A**/**Q** layer model with an alternating multilayer of **AQA** layer and **Q** layer. (a) PL spectra of **Q** unary multilayer (top) and **A**/**Q** (bottom) with multilayering up to 4L or 8L. (b) (i) The ratio and (ii) the difference of PL intensity relative to the lower layer. Red and blue bar represent the increase and the decrease in PL intensity from the lower layer, respectively. PL intensities in (b) were normalized by the simulated PL intensity of 1L of **Q**.
